# Burden of trachoma in five counties of Eastern Equatoria state, South Sudan: Results from population-based surveys

**DOI:** 10.1371/journal.pntd.0005658

**Published:** 2017-06-14

**Authors:** Angelia M. Sanders, Aisha E. P. Stewart, Samuel Makoy, Joy J. Chebet, Peter Magok, Aja Kuol, Carla Blauvelt, Richard Lako, John Rumunu, E. Kelly Callahan, Scott D. Nash

**Affiliations:** 1 Trachoma Control Program, The Carter Center, Atlanta, Georgia, United States of America; 2 South Sudan Ministry of Health, Government of the Republic of South Sudan, Juba, Republic of South Sudan; 3 The Carter Center-South Sudan, The Carter Center, Juba, Republic of South Sudan; Institut Pasteur, FRANCE

## Abstract

**Background:**

In order to decrease the prevalence of trachoma within the country, the Republic of South Sudan has implemented components of the SAFE strategy in various counties since 2001. Five counties in Eastern Equatoria state were surveyed in order to monitor progress of programmatic interventions and determine if additional rounds of Mass Drug Administration with azithromycin were needed.

**Methodology/ Principal findings:**

Five counties (Budi, Lafon, Kapoeta East, Kapoeta South and Kapoeta North) were surveyed from April to October 2015. A cross-sectional, multi-stage, cluster-random sampling was used. All present, consenting residents of selected households were examined for all clinical signs of trachoma using the World Health Organization (WHO) simplified grading system. 14,462 individuals from 3,446 households were surveyed. The prevalence of trachomatous inflammation-follicular (TF) in children ages one to nine years ranged from 17.4% (95% Confidence Interval (CI): 11.4%, 25.6%) in Budi county to 47.6%, (95% CI: 42.3%, 53.0%) in Kapoeta East county. Trachomatous trichiasis (TT) was also highly prevalent in those 15 years and older, ranging between 2.6% (95% CI: 1.6%, 4.0%) in Kapoeta South to 3.9% (95% CI: 2.4%, 6.1%) in Lafon. The presence of water and sanitation were low in all five counties, including two counties which had a complete absence of latrines in all surveyed clusters.

**Conclusions/ Significance:**

To our knowledge, these were the first trachoma surveys conducted in the Republic of South Sudan since their independence in 2011. The results show that despite years of interventions, four of the five surveyed counties require a minimum of five additional years of SAFE strategy implementation, with the fifth requiring at minimum three more years.

## Author summary

Trachoma is the leading cause of preventable blindness in the world; however, with proper interventions the disease can be controlled. Despite a paucity of experienced trachoma graders, insecurity, and difficulty accessing some villages, population-based impact surveys were conducted in five counties of South Sudan in 2015, with over 10,600 persons examined by trained and certified graders. Results showed that all five counties surveyed in Eastern Equatoria state of South Sudan face considerable trachoma burdens. The presence of water and sanitation were low in all five counties, including two counties which had a complete absence of latrines in all surveyed villages. The global community cannot expect to eliminate trachoma by 2020 if South Sudan is not provided with the human and financial resources it needs to not only understand the full extent of the disease in the country but also to implement programmatic interventions.

## Introduction

Trachoma, the leading cause of preventable blindness, is a disease caused by ocular infection with the bacterium *Chlamydia trachomatis* [1]. Over time, repeated infections can lead to conjunctival scarring of the upper eyelid, which may result in the eyelid turning inwards against the globe and cornea (entropion) and causing irritation and abrasion of the eye by the lashes (trachomatous trichiasis). If left untreated, scarring and opacification of the cornea can occur leading to irreversible blindness in the eye. In order to address the public health burden of trachoma globally, the World Health Organization (WHO) has endorsed the SAFE strategy: Surgery for those with trichiasis, mass distribution of Antibiotics to reduce infection, and promotion of Facial cleanliness and Environmental improvement through a focus on latrine use as a way to decrease modes of transmission. According to WHO recommendations, the full SAFE strategy is warranted at the district level if the prevalence of trachomatous inflammation-follicular (TF) is greater than 10% in children ages one to nine years [2].

Previous prevalence surveys conducted across many counties in South Sudan between 1999 and 2010 demonstrated the need for the SAFE strategy throughout assessed regions [3–7]. Furthermore, some suspected endemic areas of the country still require baseline mapping to understand the magnitude of the prevalence of trachoma and the interventions needed. Despite years of conflict and weak infrastructure, South Sudan has implemented components of the SAFE strategy in various counties since 2001 [8]. Since 2007, SAFE activities have been implemented in five Eastern Equatoria counties, with all five receiving at least four rounds of MDA, multiple surgical campaigns, and health education activities. The Ministry of Health, Republic of South Sudan (MoH-RSS), with the support of The Carter Center, implemented population-based trachoma surveys in five counties to determine if full SAFE strategy interventions were still warranted.

## Methods

### Ethics statement

Ethical clearance was received from the Ethical Committee of the Ministry of Health of South Sudan and the Emory University Internal Review Board (IRB 079–2006). Due to high illiteracy among the population and logistical constraints of written consent forms, IRB approval was obtained for verbal informed consent to be collected from all participants and recorded electronically. For those under 16 years of age, verbal consent from a parent or guardian was required. Participants were free to withdraw consent at any time without consequence.

### Setting

**Fig 1:**
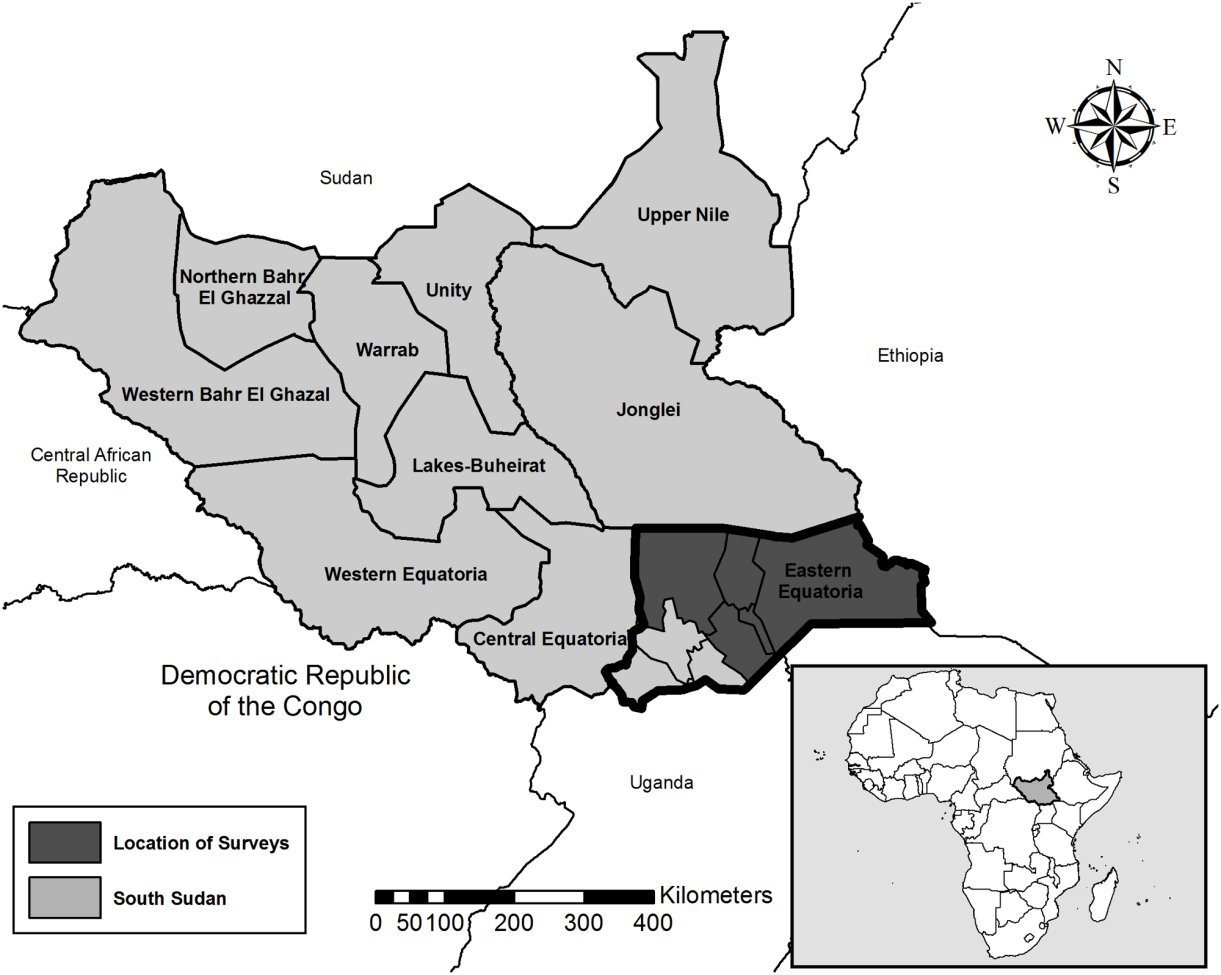
Trachoma survey location, Eastern Equatoria State, South Sudan.

Between April and October 2015, we conducted population-based surveys in the following five counties: Budi, Lafon, Kapoeta East, Kapoeta North, and Kapoeta South (Fig 1). Administratively, South Sudan is divided into states, counties, payams, bomas, and villages. Counties are the equivalent of a district and are the level at which trachoma activities are implemented. In 2015, the government of South Sudan re-defined and renamed state borders; however, since the surveys were conducted before this time, this report refers to the original political boundaries of Eastern Equatoria state. Of the baseline surveys conducted in Eastern Equatoria state between 2001 and 2004 [3], the populations in Kimotong and Narus payams were examined. Kimotong payam, located in Budi county, had a TF prevalence of 40% (CI: 34.6%, 45.7%) in children ages one to nine years and TT prevalence in persons 15 years and above of 17% (CI: 14.6%, 19.6%), while Narus payam, located in Kapoeta East county, had a TF prevalence of 35% (CI: 31.6%, 39.3%) and TT prevalence of 6.3% (CI: 4.7%, 8.2%). For programmatic purposes, Kimotong’s prevalence data was applied to all of Budi county, while Narus’ data was applied to Kapoeta East, Kapoeta North, and Kapoeta South counties. Due to Lafon’s proximity to these counties, it was assumed to be equally endemic.

### Sampling

To estimate TF prevalence among children ages one to nine years with 95% confidence, a sample size was calculated using an assumed TF prevalence in this age group of 10% ±5.0% precision, and a design effect of five [9]. We assumed a 15% non-response rate, five people per household and that 29% of the population was one to nine years old [4]. Based on these assumptions, we estimated that 794 children ages one to nine years would need to be surveyed in each county to obtain accurate prevalence estimates. To achieve this sample size, we surveyed 20 clusters, with a cluster consisting of one village of 30 households. Budi and Lafon surveys were conducted in April and May 2015, while Kapoeta East, Kapoeta North, and Kapoeta South were surveyed August to October 2015. Based on a higher than expected TF prevalence and a lower mean household size in Budi and Lafon counties (4.6%), survey assumptions were modified in order to estimate a TF prevalence of 20% ± 5%, which led us to survey 25 clusters of 35 households for the three remaining counties of Kapoeta East, Kapoeta North, and Kapoeta South.

For all five surveys, the same multi-stage cluster-random sampling method was employed to assure a known, non-zero probability of selection for every individual in each county. In the first stage, clusters were systematically selected from a geographically ordered list using probability proportional to population size for each county based on village population estimates provided by the Ministry of Health South Sudan Trachoma Control Program and the South Sudan Guinea Worm Eradication Program. Villages with a population <100 persons or towns >5,000 persons were excluded from the sampling frame [10]. In the second stage, households were selected randomly using a sketch map and segmentation method [11]. Households within a cluster were selected with equal probability, and all present consenting household members were examined for all signs of trachoma [12].

### Training

Data recorders were selected from the counties in which data was being collected to ensure they were able to communicate with the survey participants in their local dialect. All recorders underwent a six-day training concerning how to use electronic tablets to collect data, conduct interviews, and randomly select households and individuals to be interviewed following the standard protocol. Following training, all recorders were required to pass an examination on their data collection and interview skills in order to participate on the survey team.

Trachoma graders were selected from a pool of currently practicing ophthalmic clinical officers working in tertiary eye care facilities throughout South Sudan. Grader training consisted of in-class and field practice using the WHO simplified grading system [12]. Each grader was required to pass both an in-class slide test and a field reliability exam with a score of 84% agreement and ≥0.70 kappa score against the consensus grade of the three grader trainers. Seven of the 10 trainees achieved the required agreement, and the top six were selected to participate in data collection.

### Data collection

Each data collection team consisted of a trachoma grader, a data recorder, and driver, with one supervisor responsible for two teams. All residents of selected households were enumerated regardless of their presence and/or willingness to be examined. All verbally consenting persons living within the household were interviewed in their local language and examined for all five signs of trachoma, as defined by the WHO simplified grading scheme [12] using a 2.5X loupe and a flashlight. Graders also observed the face of each child, ages one to nine, to determine if their face was unclean, defined as the presence of ocular or nasal discharge [13]. Any participant that was found to have TF or trachomatous inflammation-intense (TI) was provided with tetracycline eye ointment. Those who were found to have TT were registered and encouraged to undergo TT surgery when the next surgical campaign was held in their county. Survey teams made one attempt to follow-up at the end of the day to examine children ages one to nine years who were absent during the examination process by returning to the child’s house. Households that were empty were not replaced by another household.

### Household interview

Structured questionnaires were used to interview a representative from each household, with special preference to interviewing female caregivers, who in this cultural context are the primary caregivers to children and are responsible for household chores such as fetching water. The household interview included questions regarding: demographics, latrine ownership and use, primary type of water source used during the dry season, distance to water source, face washing practices with children, cattle keeping, and ownership of radios and mobile phones. In order to clarify the type of water source, pictures of various water sources were shown to the respondent. An improved water source was defined as piped water into dwelling, a public tap, a protected dug well, or a protected spring [14]. When a latrine was present, recorders and/or graders verified use through asking the respondent if they used the latrine and directly observing signs of use, including a worn path to the latrine, presence of fresh feces in the latrine [15], and presence of materials for anal cleansing and/or hand washing.

### Data entry and analysis

All data were collected electronically on Samsung Galaxy tablet computers loaded with custom built survey software, Swift Insights [16]. Data were downloaded from tablet computers periodically by field supervisors. While in the field, supervisors reviewed the data collected by recorders and documented errors encountered during data collection to assist in data cleaning. Sampling weights were calculated as the inverse of the probability of selection at both stages of sampling. Confidence intervals were calculated using Taylor linearization through *svy* survey procedures in Stata 13.1 (StataCorp LP. [http://www.stata.com]) taking into account the multi-level structure of the sampling. All reported percentages with confidence intervals are weighted. Post-stratification weighting using five-year age-sex bands from the survey census population was used when estimating the prevalence of trachomatous scarring (TS), TT, and corneal opacity (CO) among the whole population and among those 15 years and older to account for systematic missingness among older males in this population. The missingness of males was perceived to be an issue because women carry an increased burden of TT compared to men [17]. All statistical analysis was conducted using Stata 13.1.

## Results

**Fig 2:**
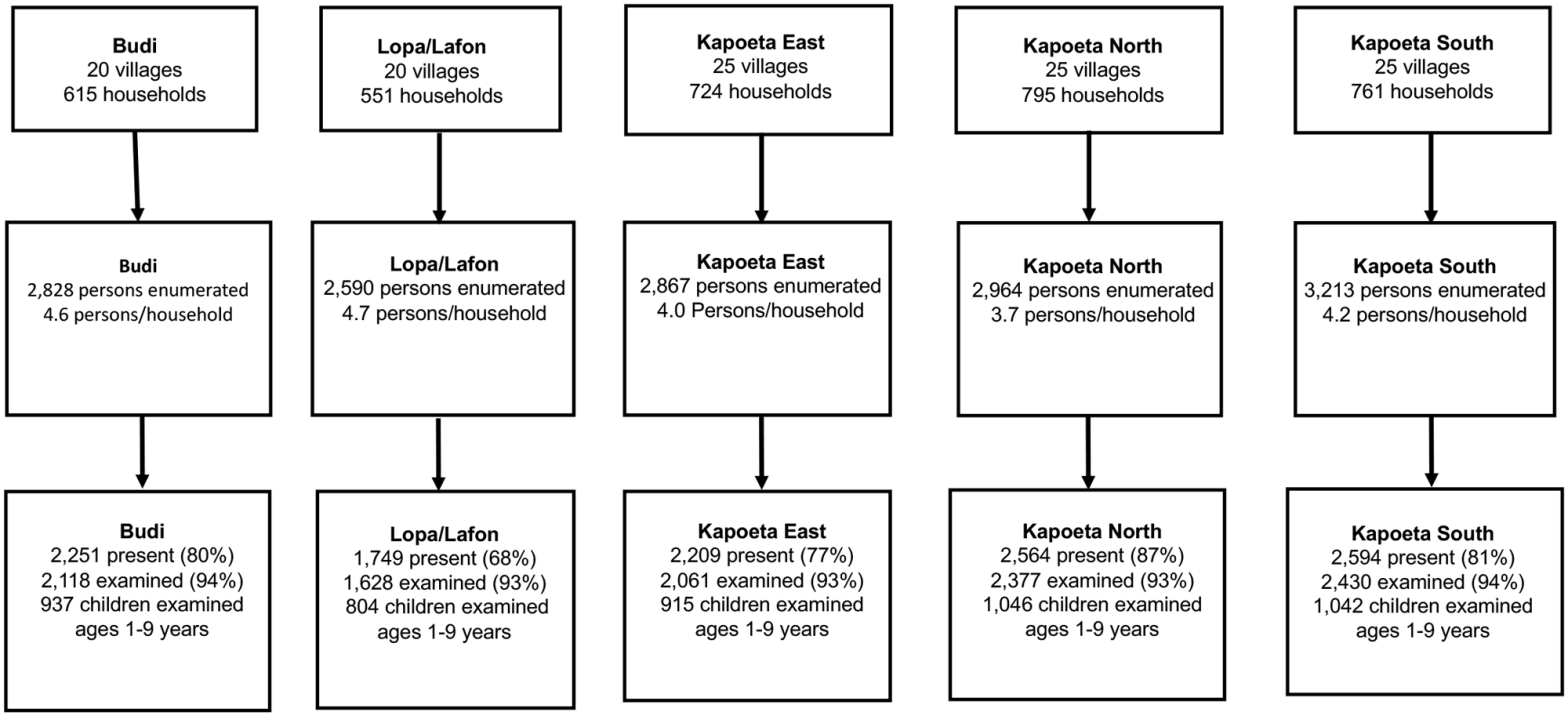
Survey population by county, Eastern Equatoria State, South Sudan 2015.

In five counties of Eastern Equatoria State, a total of 14,462 individuals in 3,446 households were enumerated, and of these 11,367 (78.6%) were present at the time of the survey (Fig 2). Among present household members, 10,614 (93.4%) consented to the examination and were included in the analyses. Among children ages one to nine years enumerated, 4,744/5,126 (92.6%) were present and examined. In the examined population with complete sex data, 6,580/10,596 (62.1%) were female and 4,016/10,596 (37.5%) were male, while among children one to nine years 2,344/4,735 (50.5%) were female and 2,391/4,735 (49.5%) were male. Eighteen individuals were missing data on sex and three individuals were missing data on age. The mean age of study for participants was 18.8 years, which was statistically significantly lower than the mean age of individuals not present at the time of the survey (22.2 years; P≤0.0001). The respondent provided demographic information about absent household members, and anecdotal evidence at the time of the survey was provided that men were away at temporary cattle camps.

Indicators of water, sanitation, and hygiene were low within all five counties (Table 1). The prevalence of an improved primary source [14] for water ranged from 1.2% (95% CI: 0.4%, 4.0%) in Lafon to 11.7% (95% CI: 3.0%, 36.4%) in Kapoeta East. In all three Kapoeta counties, nearly one third of households reported that it took them greater than 60 minutes to fetch water and return home. The county with the highest household latrine coverage was Kapoeta South (10%; 95% CI: 4.0%, 22.7%), which is the location of the major town for all three Kapoeta counties, while in Kapoeta East and Kapoeta North, no household latrines were found by survey teams. Overall, 54.1% (95% CI: 49.7%, 58.5%) of children ages one to nine years showed signs of a clean face.

**Table 1:** Individual and household characteristics by county, Eastern Equatoria State, South Sudan 2015.

Characteristics	Budi % (95% CI)	Lafon % (95% CI)	Kapoeta East % (95% CI)	Kapoeta North % (95% CI)	Kapoeta South % (95% CI)
**Individual**					
Children ages 1–9 y with clean face (observed)	43.4 (31.2–56.4)	45.2 (39.7–50.8)	57.8 (49.3–65.9)	65.2 (56.0–73.3)	57.9 (51.3–64.2)
Children ages 5–15 y attending school	21.2 (9.9–39.7)	38.0 (25.7–52.1)	0.8 (0.2–3.3)	1.7 (0.9–3.2)	17.4 (8.6–32.1)
**Household**					
Caregivers washing children’s faces					
Never	0.2 (0.0–0.8)	1.5 (0.6–3.5)	25.7 (13.1–44.3)	13.3 (4.9–31.1)	2.7 (1.3–5.6)
Every few days	0.0	0.7 (0.2–2.7)	7.4 (4.3–12.6)	12.8 (7.9–20.2)	4.1 (1.2–13.2)
Once a day	55.6 (43.7–66.8)	31.2 (23.0–40.8)	25.6 (17.8–35.5)	28.4 (20.2–38.3)	29.4 (17.5–45.0)
Twice a day	40.3 (29.8–51.8)	56.0 (48.6–63.0)	31.2 (22.3–41.8)	38.2 (27.8–49.8)	45.2 (31.0–60.3)
More than twice a day	3.9 (1.7–9.1)	10.7 (5.5–19.4)	10.0 (6.2–15.9)	7.4 (4.6–11.5)	18.6 (10.9–29.8)
Presence of latrine (observed)	9.9 (2.8–29.6)	0.4 (0.1–2.0)	0.0	0.0	10.0 (4.0–22.7)
Improved primary source of water	1.2 (0.4–4.1)	1.2 (0.4–4.0)	11.7 (3.0–36.4)	11.2 (3.2–32.8)	7.3 (2.7–18.3)
Time to collect water					
<30 min	55.8 (36.3–73.6)	46.7 (29.6–64.7)	31.0 (16.0–51.5)	18.4 (8.0–37.1)	26.6 (15.3–42.1)
30–60 min	33.1 (20.0–49.5)	46.7 (30.5–63.6)	38.4 (22.3–57.5)	52.2 (34.3–69.6)	34.9 (17.5–57.5)
>60 min	11.1 (4.1–26.5)	6.6 (2.8–14.9)	30.6 (16.0–50.6)	29.4 (15.1–49.4)	38.5 (20.0–61.0)
Cattle ownership	17.0 (11.0–25.5)	28.4 (17.1–43.2)	99.9 (99.2–100.0)	100	78.4 (57.6–90.7)
Cattle kept < 20 meters from house	43.2 (22.3–67.0)	44.0 (28.6–60.7)	19.6 (12.9–28.7)	39.7 (25.9–55.4)	58.4 (41.8–73.2)
Radio ownership	3.0 (1.2–7.5)	9.4 (6.3–13.8)	0.2 (0.0–1.5)	0.4 (0.1–1.4)	5.1 (2.5–10.1)
Mobile phone ownership	13.8 (5.5–30.7)	15.6 (10.3–23.0)	0.9 (0.3–2.2)	1.0 (0.5–2.0)	22.6 (11.9–38.8)
Any adult education	22.0 (12.1–36.6)	30.6 (23.1–39.4)	1.2 (0.4–3.5)	0.7 (0.3–2.1)	11.9 (5.5–23.9)

Note: Confidence Intervals are in (). Clean face was observed and defined as: absence of nasal and ocular discharge. Improved water source defined as: piped water into dwelling, a public tap, a protected dug well, or a protected spring. Any adult education included: at least one year of primary school or higher, any technical training, or university education.

**Fig 3:**
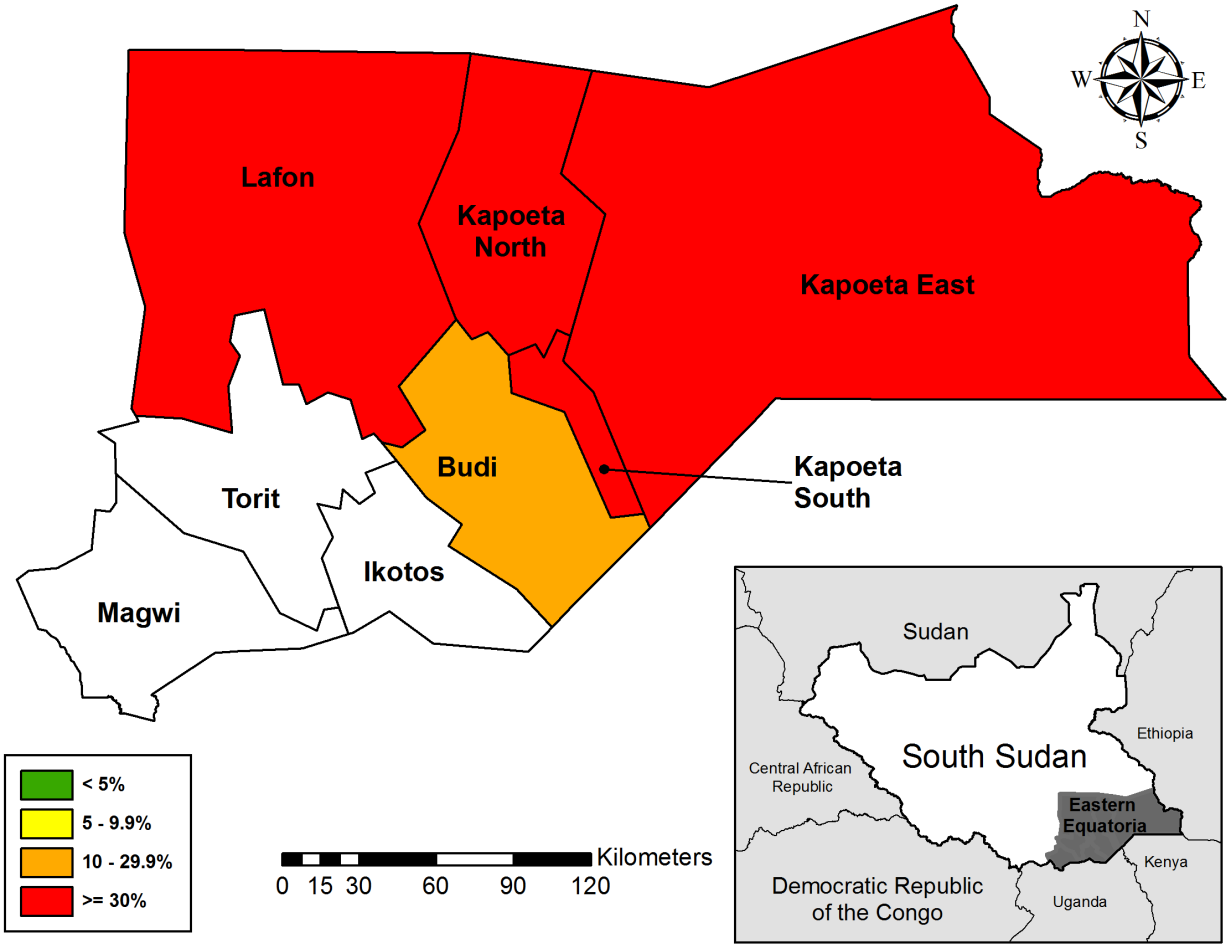
Prevalence of TF in children ages 1–9 years in 5 counties of Eastern Equatoria State, South Sudan 2015.

**Fig 4:**
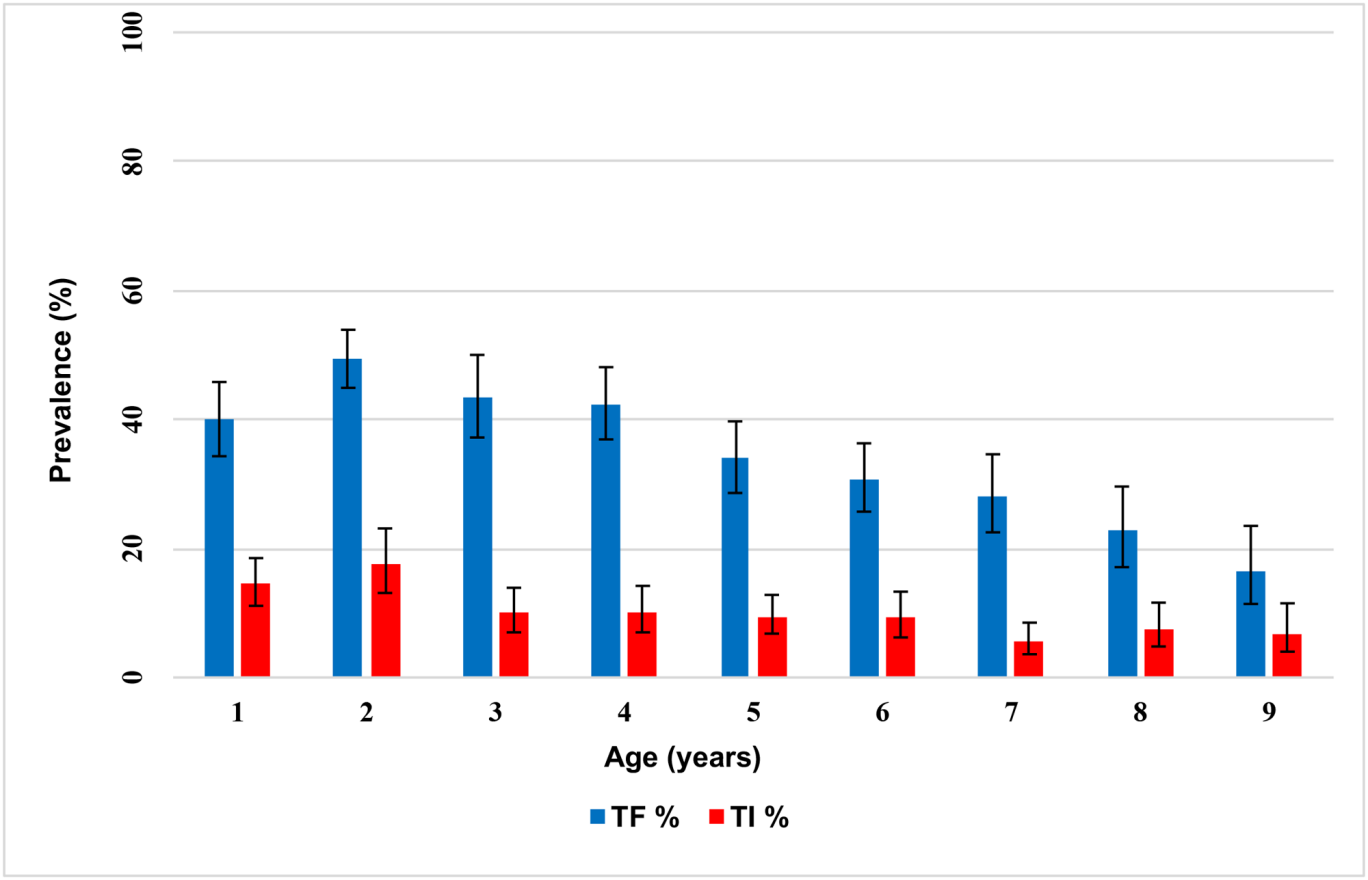
Age-specific prevalence of TF and TI among children ages 1 to 9 years.

The prevalence of TF in children ages one to nine years was 17.4% (95% CI: 11.4%, 25.6%) in Budi, 35.3% (95% CI: 28.7%, 42.5%) in Lafon, 47.6% (95% CI: 42.3%, 53.0%) in Kapoeta East, 39.7% (95% CI: 32.3%, 47.6%) in Kapoeta North, and 30.1% (95% CI: 23.4%, 37.9%) in Kapoeta South counties (Table 2). All five of these counties were therefore over the 10% TF threshold and require the full SAFE strategy, including the continuation of MDA with azithromycin (Fig 3). Budi had the lowest prevalence of TI, 5.8% (95% CI: 2.5%, 12.7%), and Kapoeta East had the highest prevalence of TI, 15.4% (95% CI: 11.7%, 20.0%). There were no statistically significant differences between males and females ages one to nine years for the prevalence of TF (35.6% vs. 36.5%; P = 0.58) or TI (10.2% vs. 10.8%; P = 0.62). Within this age group, both TF and TI were higher among younger children (< 7 years) and lower in those children between seven and nine years old (Fig 4). The prevalence of TF in participants age 15 years and older was low in all counties, ranging from 0.6% (95% CI: 0.2%, 2.1%) in Budi to 1.9% (95% CI: 0.9%, 4.0%) in Lafon.

**Table 2:** Prevalence of clinical signs of trachoma in five counties of Eastern Equatoria State, South Sudan 2015.

Clinical Sign	Budi % (95% CI)	Lafon % (95% CI)	Kapoeta East % (95% CI)	Kapoeta North % (95% CI)	Kapoeta South % (95% CI)
**TF**, ages 1–9 y	17.4 (11.4–25.6)	35.3 (28.7–42.5)	47.6 (42.3–53.0)	39.7 (32.3–47.6)	30.1 (23.4–37.9)
**TI**, ages1-9 y	5.8 (2.5–12.7)	12.2 (7.5–19.2)	15.4 (11.7–20.0)	8.1 (5.7–11.5)	5.3 (2.7–10.1)
**TF and/or TI**, ages 1–9 y	20.2 (13.0–30.1)	37.9 (30.4–46.1)	50.6 (45.0–56.2)	41.3 (33.7–49.3)	31.8 (23.9–40.9)
**TS**, ages ≥15 y	2.9 (0.9–8.6)	6.6 (3.5–12.1)	3.5 (2.3–5.1)	2.8 (1.9–4.1)	3.5 (1.5–7.8)
**TS**, all ages	1.6 (0.5–4.7)	3.4 (2.0–5.9)	1.7 (1.2–2.6)	1.5 (1.0–2.3)	1.9 (0.9–4.1)
**TT**, ages ≥15 y*	3.2 (2.0–2.0)	4.7 (3.0–7.3)	4.3 (3.0–6.1)	3.9 (2.8–5.5)	3.0 (2.0–4.5)
**TT**, ages ≥15 y	2.7 (1.6–4.5)	3.9 (2.4–6.1)	3.7 (2.6–5.3)	3.3 (2.3–4.6)	2.6 (1.6–4.0)
**TT**, all ages	1.4 (0.8–2.3)	2.6 (1.4–4.8)	1.9 (1.3–2.8)	1.8 (1.3–2.5)	1.3 (0.8–2.0)
**CO**, ages ≥15 y	1.1 (0.3–3.5)	2.5 (1.4–4.4)	1.7 (0.9–3.0)	1.3 (0.8–2.3)	0.9 (0.4–1.9)

TF = trachomatous inflammation-follicular; TI = trachomatous inflammation-intense; TS = trachomatous scarring; CO = Corneal Opacity; 95% Confidence Intervals are in ().

* Estimates without post-stratification weights applied.

**Fig 5:**
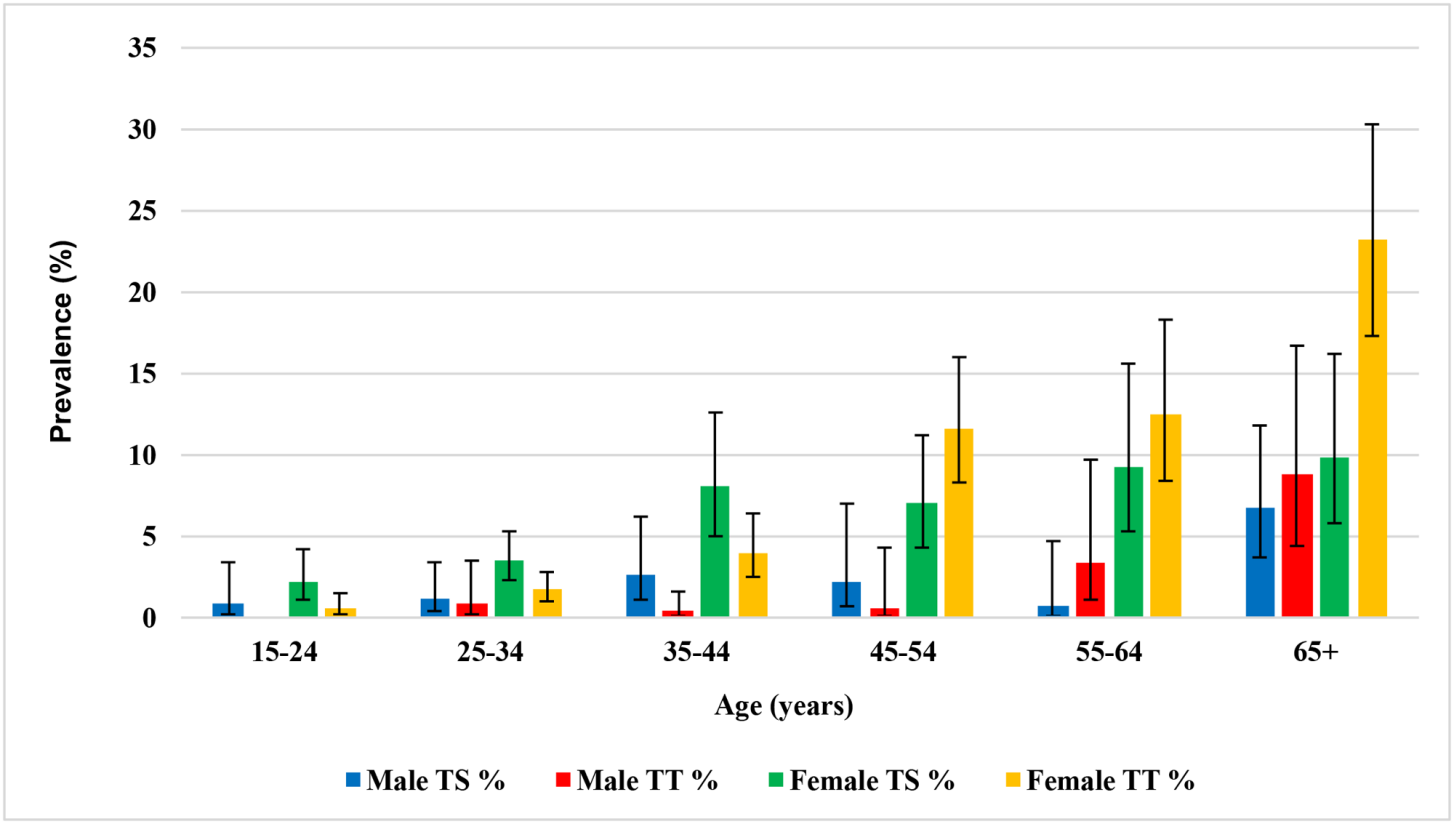
Age-specific prevalence of TS and TT among adults 15 years and older.

The prevalence of TS among participants 15 years and older ranged from 2.9% (95% CI:0.9%, 8.6%) in Budi to 6.6% (95% CI: 3.5%, 12.1%) in Lafon. Among this age group, the prevalence of TS was higher in females than in males (males = 1.9% vs. females = 4.9%; P = 0.0001) and increased with age (P≤0.0001) (Fig 5).

**Fig 6:**
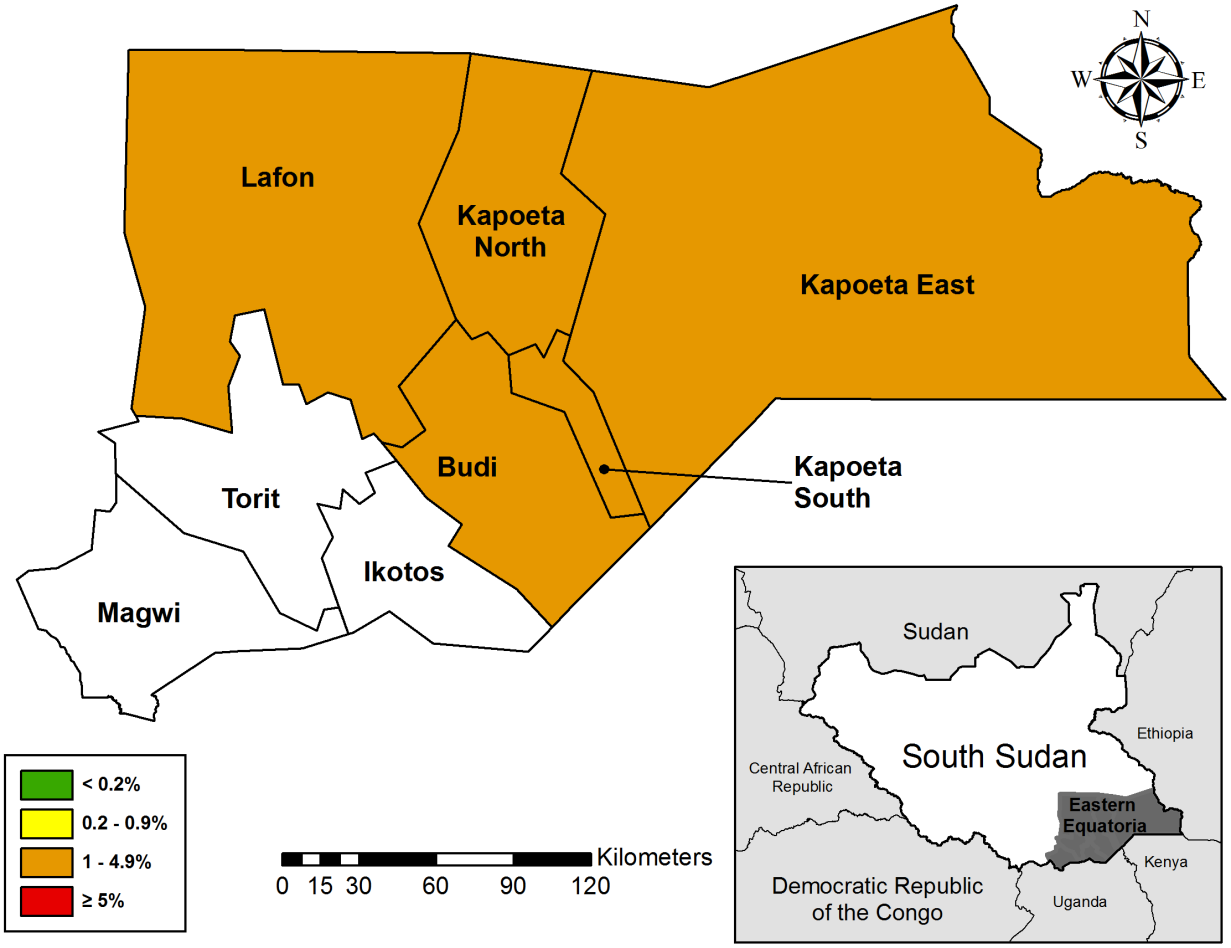
Prevalence of TT in participants ages 15+ in 5 counties of Eastern Equatoria State, South Sudan 2015.

TT was observed across the age spectrum; however, only nine cases were detected among children younger than 15 years. The prevalence of TT in adults 15 years and above ranged from 2.6% (95% CI: 1.6%, 4.0%) in Kapoeta South to a high of 3.9% (95% CI: 2.4%, 6.1%) in Lafon county. Post-stratification weighting, used to account for systematic missingness among adult males 15 years and older, caused a reduction of TT estimates for all five counties. Similar to TS, TT was more prevalent in females than males in this age group (males 1.3% vs. females = 4.6%; P≤0.0001) and increased with age (P≤0.0001). All five of the counties exceed the <0.2% elimination target set by the WHO [2] (Fig 6). Among participants 15 years and above found to have TT (n = 182), 28 (21.6%, 95% CI:14.6%, 30.9%) reported having had surgery and 50 (29.8%, 95% CI: 20.6%, 41.0%) were observed by the grader as having signs of epilation. Among those with TT who reported not having had surgery, five participants (3.6% 95% CI: 1.4%, 9.1%) reported refusing surgery and 110 participants (96.4%, 95% CI: 90.9%, 98.6%) reported not knowing about or not being offered surgery.

## Discussion

The results of these population-based impact surveys demonstrate that trachoma remains a major public health problem in these areas of South Sudan. The prevalence of TF among children ages one to nine years was greater than 10% in all five counties, with four of five above 30%; therefore, all counties require the continuation of MDA for trachoma control. The prevalence of TT in those 15 years and older was above WHO thresholds in all counties, demonstrating the need for continued surgical interventions to avoid preventable blindness. Water and sanitation factors, such as presence of latrines, access to water, and child facial cleanliness, were low in all counties, suggesting that the environmental and behavioral conditions in this part of the country enable the transmission of the causative agent of trachoma.

Despite years of civil unrest prior to South Sudan’s independence from Sudan in 2011 and the recent ethnic conflict beginning in December 2013, South Sudan has carried out components of the SAFE strategy in varying degrees since 2001. Within the five counties surveyed, SAFE activities began in 2007 in coordination with the South Sudan Guinea Worm Eradication Program. Budi and Lafon received seven rounds of MDA, with the last MDA conducted in 2013. Kapoeta North received five rounds of MDA, with the last MDA in 2011, and both Kapoeta South and Kapoeta East received four rounds of MDA, with the last MDA completed in 2010. Despite these reported rounds, according to programmatic records, drug coverage varied between counties and years, with poor coverage often coinciding with local insecurity that prevented distribution teams from accessing all villages. Surgical campaigns were conducted at various points throughout the 2007 to 2013 time period. Some water provision was provided through the partners of the South Sudan Guinea Worm Eradication Program’s infrastructure, and health education was conducted during MDA and surgical campaigns.

Previous baseline and impact surveys showed that the prevalence of trachoma varied throughout the country, with low levels of trachoma detected in Western Equatoria [5], Northern Bahr-el-Ghazal [6], and Central [18] states. Hyper endemic levels of trachoma were detected in the Greater Upper Nile region of Jonglei, Upper Nile, and Unity states [3, 7], and Eastern Equatoria state [3]. The baseline survey in Eastern Equatoria state was conducted in smaller evaluation units (payams) and is therefore difficult to directly compare to the current results, which were conducted at the county level. At baseline, Kimotong payam in Budi county was hyperendemic for TF (40%) and TT (17%) in 2004. The current data from Budi county as a whole demonstrates that lower levels of these key indicators may reflect successful SAFE implementation in the county. Narus payam in Kapoeta East county was also hyperendemic for TF (35.4%) and TT (6.3%) in 2004. The current county-level data from Kapoeta East county demonstrates the county remains hyperendemic. This is likely due to the county only receiving four rounds of MDA, with the last being in 2010, a five-year gap between MDA and the 2015 impact survey. As the WASH related data shows, there is limited presence of latrines and access to water. The other three counties (Lafon, Kapoeta South, and Kapoeta North) cannot be compared, due to baseline data not being collected from those counties.

Moving forward, all components of the SAFE strategy need to be implemented. Four of the five counties require five rounds of MDA; however, given that all five counties border each other and consist of nomadic and semi-nomadic communities, in addition to the burden of trachoma in surrounding countries and parts of South Sudan, the trachoma program should consider, at minimum, five annual rounds of MDA for all five counties. Each MDA should aim for greater than 80% coverage. In order to do this, MDAs should be conducted during months of the year when cattle herding members of the community are staying within the village.

In regards to surgery, there is need for sustained surgical interventions in all five counties. However, this can only be achieved if there is also a corresponding investment in increasing ophthalmic services in the country at all administrative levels. According to an assessment of eye care workers in 21 countries of sub-Saharan Africa in 2011, less than one ophthalmologist per one million population were present in South Sudan, thereby making it the country with the lowest ratio of ophthalmologists to population in the countries surveyed and revealing the need for more ophthalmic professionals in the country [19]. The current surveys found that prevalence of TT in all five counties was ≥2.8% among adults 15 years and older. This estimate takes into account the fact that older women are more likely to be examined in household surveys of this nature. As demonstrated in Table 2, failing to account for this fact in Eastern Equatoria state would have led to increased TT prevalence estimates in all five counties. The surveys demonstrated that epilation was an existing practice in these areas, and that lacking immediate surgical services, it may be possible to promote proper epilation practices within these populations [20]. The 96.4% of TT cases identified as not being offered surgery highlights the challenge in accessing patients and reinforces the need to scale up training of TT surgeons, the need for continual implementation of surgical camps in remote areas of each county, and the need for increased efforts to inform community members about surgical campaigns so that they may attend. Lastly, 21.6% of participants with TT reported having had surgery previously. This suggests a fairly high level of recurrence; therefore, future plans should include accommodating these complicated cases.

Facial cleanliness and environmental improvement education campaigns will need to be an integral part of the trachoma program; however, there will be immense challenges as access to water and sanitation services are limited. Based on this population-based data, these five counties have not achieved basic minimum standards for clean water and sanitation, with at least 88% of the population lacking access to an improved source of drinking water and at least 90% lacking latrine access, services which are not only important for preventing trachoma, but also in reaching the Sixth Sustainable Development Goal of access to water and sanitation for all by 2030 [21]. Within South Sudan, education campaigns will need to be developed within the context of communities that have low school attendance (less than 1% in Kapoeta East county), low adult education (ranging from 0.7% to 30.6%), and limited phone and mobile radio ownership.

The Trachoma Control Program of South Sudan overcame many challenges while implementing these surveys, most notably a paucity of experienced trachoma graders, insecurity, and difficulty accessing some villages. In order to ensure the training of highly reliable trachoma graders, the MoH-RSS requested assistance from its neighboring endemic countries, and Ethiopia, Sudan, and Uganda responded by providing experienced grader trainers to South Sudan. This successful cross-border training activity provided South Sudan with much needed technical assistance. Insecurity in the surveyed counties was addressed through pre-survey meetings with appropriate representatives from the national, state, payam, and village levels. Because of this pre-survey communication, communities were less suspicious of the survey teams’ motives and activities when the survey teams arrived. Additionally, armed security escorts were organized for main transport corridors that had reported security incidences. In Budi and Lafon, villages are often located along hill and mountain ridges in order to provide security to the communities. These locations resulted in teams having to hike long distances to reach villages, which extended the duration of the survey. Despite these challenges, the teams reached all randomly selected clusters and households and examined the necessary minimum number of required individuals.

Outside of strengthening the national Trachoma Control Program’s ability to implement SAFE activities, partners should provide South Sudan with the resources needed to conduct more prevalence surveys. Many counties still require baseline “mapping”, with most of these assumed to be highly endemic; thereby, needing many years of SAFE implementation. Knowing the true extent of trachoma in the country will better enable the Ministry of Health to advocate for resources and plan interventions to help avoid preventable blindness. The five counties surveyed border trachoma-endemic countries. According to the Global Trachoma Atlas [22], to the East they border parts of Ethiopia with a reported TF prevalence between 10–29.9% and Kenya with a reported TF prevalence over 30% [23], and to the South, these counties border areas of Uganda with a TF prevalence between 5–9.9%. Successful trachoma elimination programs in those three countries will likely depend on continuing progress in South Sudan, given the cross-border movement that is common with cattle herding communities and the continued displacement of people, internally and internationally, due to ongoing conflict. A strategy which brings together these Ministries of Health to target these cross-border areas is needed in order to control trachoma regionally by 2020. The global community cannot expect to eliminate trachoma by 2020 if South Sudan is not provided with the human and financial resources it needs to not only understand the full extent of the disease in the country, but also to implement the various components of the SAFE strategy.

## Conclusion

Despite insecurity, lack of infrastructure, and limited in-country trained eye care personal, South Sudan, with leadership in the Ministry of Health, has shown its desire to eliminate trachoma through its willingness to implement the SAFE strategy and conduct impact surveys under challenging circumstances. All five counties surveyed in Eastern Equatoria state of South Sudan face considerable trachoma burdens. TF was above 30% in four counties and 17% in one county, showing the need for multiple years of MDA. TT was above the WHO threshold of 0.2% in those 15 years and above in all five counties, thereby requiring surgical interventions. The presence of water and sanitation were low in all five counties, including two counties which had a complete absence of latrines in all surveyed clusters. These survey results will enable the national program to use valuable, updated data to better plan programmatic activities, thereby helping the country make significant progress towards eliminating blindness from trachoma.

## Supporting information

S1 Checklist STROBE checklist. (DOC)
